# Dietary fiber may benefit chondrocyte activity maintenance

**DOI:** 10.3389/fcimb.2024.1401963

**Published:** 2024-05-13

**Authors:** Ying Wu, XiangJie Li, Hao Meng, Ying Wang, Peng Sheng, YongNing Dong, Ju Yang, BingQian Chen, XueSong Wang

**Affiliations:** ^1^ Department of Orthopedics, Affiliated Hospital of Jiangnan University, Wuxi, Jiangsu, China; ^2^ Department of Nutrition, Affiliated Hospital of Jiangnan University, Wuxi, China; ^3^ Wuxi School of Medicine, Jiangnan University, Wuxi, Jiangsu, China; ^4^ Department of Orthopaedics, Changshu Hospital Affiliated to Soochow University, First Peoples’ Hospital of Changshu City, Changshu, Jiangsu, China

**Keywords:** osteoarthritis, gut microbiota, SESN2, SCFAs, fiber dietary

## Abstract

The understanding of the link between the gut-bone axis is growing yearly, but the mechanisms involved are not yet clear. Our study analyzed the role of Sestrin2 (SESN2)pathway in the gut-bone axis. We established an osteoarthritis (OA) model in Sprague-Dawley (SD) rats using the anterior cruciate ligament transection (ACLT) procedure, followed by a dietary intervention with varying levels of dietary fiber content for 8 weeks. By 16S rRNA sequencing of the gut microbiota, we found that high dietary fiber (HDF) intake could significantly increase the Bacillota-dominant gut microbiota. Meanwhile, enzyme linked immunosorbent assay (ELISA) and histological analysis showed that intervention with HDF could reduce the degree of bone and joint lesions and inflammation. We hypothesize that HDF increased the dominant flora of Bacillota, up-regulated the expression of SESN2 in knee joint, and reduced gut permeability, thereby reducing systemic inflammatory response and the degree of bone and joint lesions. Therefore, the present study confirms that changes in gut microbiota induced by increased dietary fiber intake delayed the onset of OA by promoting up-regulation of SESN2 expression at the knee joint to maintain chondrocyte activity and reduce synovial inflammation.

## 1 Introduction

Osteoarthritis (OA) is a prevalent degenerative joint disease that is characterized by cartilage deterioration, joint inflammation, and impaired joint function (Loeser et al., 2012; Yao et al., 2023). Recent research advancements have primarily focused on understanding cartilage degeneration and synovial inflammation in OA (Kapoor et al., 2011; Paesa et al., 2023). Previous studies have implicated several signaling pathways, including the Wnt/beta-catenin (Wnt/β-catenin), Nuclear factor-κB (NF-κB), Growth factor transforming growth factor β and bone morphogenetic proteins (TGFβ/ΒΜP), and The fibroblast growth factor (FGF) pathways, in the development of OA (Yao et al., 2023). A comprehensive investigation of these pathways has highlighted the critical importance of key regulators such as Adenosine monophosphate (AMP)-activated protein kinase (AMPK), mechanistic target of rapamycin kinase (mTOR), and Runt-related transcription factor 2 (RUNX2). SESN2, an essential member of the SESN family, is a highly conserved stress-inducible protein that is susceptible to DNA damage, mitochondrial dysfunction, and oxidative stress; numerous studies have confirmed that SESN2 plays a critical role in the vital regulatory pathways mentioned above (Hou, 2015). In addition, previous studies have demonstrated its crucial role in enhancing autophagy (**Table 1**), reducing ROS aggregation, slowing cell growth and up-regulating cellular activity (Budanov, 2010). On this basis, the researchers established that the expression of SESN2 was significantly suppressed in the presence of OA, primarily through the regulation of mTOR activity that affects the function of chondrocytes, a finding that proves its essential role in OA (Shen et al., 2017).

**Table 1:** RT-PCR primer sequences for tight junction molecules in colonic tissue.

	Forword(5’-3’)	Reverse(5’-3’)
Occludin	ACT CCT CCA ATG GAC AAG TG	CCC CAC CTG TCG TGT AGT CT
ZO-1	CCA CCT CTG TCC AGC TCT TC	CAC CGG AGT GAT GGT TTT CT
β-actin	GCT GAG AGG GAA ATC GTG CGT G	CCA GGG AGG AAG AGG ATG CGG

^1^RT-PCR, Reverse transcription polymerase chain reaction; ZO-1, zonula occludens.

The level of SESN2 expression influences the direction of important signaling pathways in organisms, but it also has been reported that its expression is susceptible to changes in the composition of the gut microbiota. In studies exploring the effects of exercise on gut microbiota composition, we found that exercise promotes an increase in butyrate-producing fecal bacteria, and butyrate production activates the butyrate-SESN2/CRTC2 pathway resulting in improved lipid metabolism (Yu et al., 2019). In contrast, ablation of SESN2 masked the benefits of exercise on gut microbiota (Yu et al., 2023). Factors that influence the composition of the gut microbiota, such as exercise and aging, also influence the development of OA, and SESN2 has been implicated in all of these factors, perhaps as a central mediator linking the gut-bone axis (O'Toole and Jeffery, 2015; Cho et al., 2022).

Ceylan Tanes et al. demonstrated the significant impact of dietary fiber on gut microbiota composition and highlighted dietary fiber as the primary source of short-chain fatty acids (SCFAs) when comparing different diets (Tanes et al., 2021). Our research has revealed that dietary fiber can offer a non-invasive treatment for OA by altering the composition of gut microbiota. This approach provides a safe and convenient alternative to conventional drugs or surgical interventions. Furthermore, we have uncovered a critical role for low-grade chronic inflammation in the gut-bone axis. Schott et al. reported that gut microbiota can modulate the inflammatory response in OA by supplementing oligofructose in obese mice (Schott et al., 2018). K.H. Collins and colleagues investigated the effects of a diet with varying fat compositions on the development of OA in SD rats. Notably, they discovered that gut microbiota can exacerbate obesity-related OA, primarily through visceral fat-mediated inflammatory responses (Collins et al., 2015). Additionally, Huang et al. demonstrated that a two-strike model exacerbated surgery-induced OA in germ-free mice with low OA severity and in mice with metabolic syndrome (Huang et al., 2020). This highlights the potential of modulating gut microbiota as an intriguing approach for multifactorial synergy in OA treatment. The pathogenesis of OA is frequently linked to metabolic disturbances, such as diabetes and hypertension, which are typically associated with aging or weight gain. These conditions contribute to chronic inflammation, oxidative stress, cellular apoptosis, and cellular dysfunction, further exacerbating OA progression (Musumeci et al., 2015)

We investigated the relationship between SESN2 gene expression and OA severity following the administration of diets with varying levels of dietary fiber. The experimental results revealed that dietary fiber supplementation improved the composition of the gut microbiota, forming a floral composition dominated by the Bacillota phylum. The Bacillota-dominated gut composition was conducive to maintaining chondrocyte activity through the reduction in systemic inflammation and the promotion of increased expression of SESN2 at the joints, which delayed the onset of OA pathology. These findings provide a theoretical basis for potential interventions targeting gut microbiota and the regulation of SESN2 gene expression in OA.

## 2 Materials and methods

### 2.1 Experimental animals and study design

We utilized specific pathogen-free (SPF), six-week-old male rats in this study that were fed and housed in the Animal Center of Jiangnan University School of Medicine. The animals were maintained in a 12-hour light-dark cycle. They were divided into three groups, each consisting of eight animals. Fecal samples were collected periodically and sent for monitoring the bacterial composition. In addition, age-matched SD rats were bred, raised, and housed in the specific pathogen-free animal facility within the same institution. All experimental animals experienced the same light-dark cycle and received the same food and water. All animal experiments were approved by the Animal Care and Use Committee of Jiangnan University and adhered to the recommendations Chinese Association for Laboratory Animal Sciences Guidelines for the Care and Use of Laboratory Animals. Which underwent ACLT surgery after one week of acclimation in the barrier environment. All rats successfully underwent the modeling procedure without any mortality. Subsequently, at eight weeks of age, after an additional week of observation, the rats were divided into three groups: the normal diet (NDF) group, moderate dietary fiber (MDF) group, and high dietary fiber (HDF) group. Each group received dietary fiber in pre-specified proportions, determined by referencing established nutritional proportions from prior research on the gut-brain axis at Xuzhou Medical University (Shi et al., 2021). The specialized diets were manufactured by Synergy Bio in accordance with the following specifications. The HDF diet was characterized by a composition comprising 20% plant polysaccharides and a substantial presence of microbial carbohydrates, including corn, soy, wheat, and oats. The moderate-fiber diet mirrored the HDF diet in composition but had a reduced fiber content of 15%. Conversely, the NDF diet contained 5% fiber and exhibited diminished gut microbial accessibility.

To monitor the onset and progression of surgically-induced OA, pain assessments were conducted at two, four, six, and eight weeks post-intervention to monitor the development of OA. These assessments were conducted within the controlled barrier environment of the Animal Center of Jiangnan University School of Medicine. Fecal samples were collected during the initial, intermediate, and final stages of the intervention. Subsequently, following an eight-week experimental period, biological samples, including blood, colon, liver, knee joints, and other tissues were harvested for further analysis.

### 2.2 Histologic analysis of the colon

We uniformly preserved the distal colons from the rats for subsequent hematoxylin & eosin (H&E) staining to measure inflammatory cell infiltration and evaluate local inflammation in colonic tissues. Briefly, the distal colon was removed and fixed with 4% paraformaldehyde, then paraffin-embedded. From this, 4μm sections were prepared and stained with H&E staining. After staining, micrographs were captured using a digital camera (Zeiss, Oberkochen, Germany) mounted on a light microscope. The images were subsequently subjected to histopathologic evaluation. This evaluation utilized established scoring systems, with a focus on two parameters: the Epithelial Score (E), encompassing 0 (indicating morphologically normal epithelium), 1 (indicating loss of cup cells), 2 (indicating extensive loss of cup cells), 3 (indicating loss of crypts), or 4 (indicating extensive loss of crypts) and the Infiltration Score (I) encompassing 0 (indicating no infiltration), 1 (indicating peri-basal infiltration of the crypts), 2 (indicating infiltration reaching the muscular layer of the mucosa), 3 (indicating extensive infiltration reaching the muscular layer of mucosal thickening, with abundant edema), or 4 (indicating infiltration of submucous layers). The total histologic score was calculated as the sum of the epithelial cell and infiltration scores (total score = E+I), resulting in a range from 0 to 8 (Kim et al., 2017).

### 2.3 Gene expression analysis of tight junction molecules

The relationship between gut microbiota composition and intestinal permeability is inextricably linked. To critically assess intestinal permeability, we collected the proximal colon (approaching the anus) for RT-PCR analysis, and PCR primer sequences were obtained from previous studies. Briefly, total RNA from colon tissues was extracted using Trizol reagent (Invitrogen,Thermo Fisher,USA) according to the manufacturer’s instructions. Genomic DNA was removed using DNase I (TaKaRa, Kyoto, Japan). Reverse transcription was carried out according to the instructions for the Reverse Transcription Kit (Vazyme,NanJing,China). All samples were reverse transcribed into cDNA and quantified using a ND-2000 (NanoDrop Technologies, Thermo Fisher,USA). High-quality RNA samples with OD260/280 = 1.8~2.2 were selected for amplification. The PCR mixtures were prepared according to the instructions for the HiScript-TS 5’/3’ RACE Kit, and PCR amplification reactions were performed. The amplification process followed an initial hold step (95°C for 3 min) followed by 45 cycles of a three-step PCR process (95°C for 15 s, 60°C for 15 s, and 72°C for 30 s). The amplification reaction was carried out using a fluorescent quantitative PCR detection system from BIO-RAD (USA). In addition, the fluorescence intensity of each sample was measured at each temperature change to monitor whether the target gene was amplified. Data analysis was performed using the comparative threshold cycling (Cq) method and normalized to the expression of the housekeeping gene, β-actin, and relative to a calibrator (2^-;;Ct^) (Huang et al., 2020).

### 2.4 16S rRNA gene sequencing, bioinformatics, and statistical analysis

All 16S rRNA sequencing results involved in this study were analyzed on the Illumina sequencing platform by Macro Sequence Biotechnology Ltd (Shanghai, China). Briefly, we extracted total DNA from rat feces according to the instructions of the E.Z.N.A.^®^ Soil DNA Kit (Omega Bio-tek, Norcross, GA, U.S.). DNA concentration and purity were checked using a NanoDrop2000, followed by PCR amplification of the V3-V4 variable region using specific primers. The PCR products were purified using the AxyPrep DNA Gel Extraction Kit (Kihara Bioscience, Union City, CA, USA) and Qubit 4 (Thermo Fisher, U.S.) and then used for quantification. Purified amplicons were subjected to equimolar and contralateral sequencing on the Illumina MiSeq PE300 platform (San Diego, CA, USA). Raw reads were deposited in the NCBI Sequence Read Archive (SRA) database (PRJNA1097012). Sequences with 97% similarity were clustered using the UPARSE algorithm, and chimeric sequences were identified and removed. Each operational taxonomic unit (OTU) representative sequence was classified using the RDP classifier against the reference database SILVA138 with a minimum confidence value of 0.7. Alpha diversity was estimated using Simpson’s index (Simpson) et al. Differences in beta diversity between groups were assessed using principal coordinate analysis (PCoA) determined by permutation multivariate analysis of variance (permanova). To compare the relative abundance of different taxa, the LEfSe method was used, while KEGG analyses were performed for differences in pathway changes between groups, with P-values < 0.05 and a size effect threshold of 2.0 for the log LDA scores. Correlation analyses were performed using Spearman rank correlation analysis (Shi et al., 2021). The specific methodology for identifying bacterial species is detailed in the online supplementary Methods section.

### 2.5 Plasma analysis for inflammatory biomarkers

Plasma samples were collected using removing an eyeball approach. In our selection of inflammatory markers, we focused on those shown to be closely associated with inflammation Caused by OA in previous studies (Wang and He, 2018). We included matrix metalloproteinase 13 (MMP-13), a specific indicator of cartilage damage. For the quantification of these markers, including IL-1β, IL-10, IL-17, TNF-α, and MMP-13, we employed ELISA kits(CUSABIO, Wuhan) which were purchased from Huamei Bio (Wuhan). The samples underwent a 1:10 dilution before being subjected to analysis. The analysis was conducted using a Luminex 100 IS instrument. To ensure the integrity of our results, all samples and LPS standard dilutions were reconstituted in endotoxin-free vials (EndoGrade^®^ Glass Test Tubes) (Hyglos GmbH, Bernried, Germany).

### 2.6 Histological evaluation of cartilage and synovial samples

For histological analysis, we dissected the right knee joint of each rat, followed by fixation in 4% formalin and decalcification with 19% EDTA (9% disodium and 10% tetrasodium, pH 7.4) over a two-week period. The knee joints were positioned in the coronal plane and embedded in paraffin using a Leica embedding unit. Subsequently, 30 slides were prepared with 4-µm tissue sections obtained using a Leica microtome.

The sections underwent a series of staining procedures, including safranin O/fast green staining, H&E staining, and immunohistochemical staining for SESN2. Briefly, the prepared sections were deparaffinized, rehydrated, immersed in a fast green solution for 25 min, followed by rinsing with 1% acetic acid, then immersed in a saffron O solution (1.5%) for 30 min, dehydrated, and coverslipped. The procedure for the remaining two staining techniques was the same as above, following established protocols (Shen et al., 2017; Huang et al., 2020). To assess cartilage lesions and synovial inflammation, we utilized the ACS scoring system. All right knees were evaluated by the same assessor (AFZ) from the Pathology Department of Southeast University School of Medicine, who was blinded to the experimental groupings.

Immunohistochemical staining for SESN2 was conducted using an antibody obtained from Proteintech (WuHan, China). To quantify the number of chondrocytes in SD rats, three photomicrographs were taken at 40× magnification, ensuring that they clearly encompassed the center of the femoral condyle, with the anterior and posterior femoral condyles suitably included. Subsequently, the total number of cells in each section and the count of SESN2-positive cells were determined (Akasaki et al., 2014).

### 2.7 ACLT surgery

All ACLT procedures were performed by a single surgical specialist under sterile conditions. Male SD rats (320-360 g) were anesthetized by inhaling 2% isoflurane until the end of the procedure. The right knee joint was shaved and sterilized with a povidone-iodine solution. A vertical incision of approximately one cm was made at the medial condyle of the right knee to mobilize the skin and expose the patellar tendon. The knee was exposed using a medial parapatellar approach. The patella was semi-dislocated laterally to expose the anterior cruciate ligament. The anterior cruciate ligament was transected with micro scissors and the knee was fully flexed. Caution was taken to prevent damaging the surrounding cartilage or other structures. The anterior laxity of the joint was verified manually via the anterior drawer test to confirm that the ACL was completely severed. Thereafter, the joint was irrigated with sterile saline. The peripatellar bursa incision and skin incision were then closed using 5-0 Vicryl pure braided absorbable sutures (Ethicon, UK) (Glasson et al., 2007).

### 2.8 Von Frey test

For habituation, mice were placed on an elevated wire grid within the testing chambers, where they acclimated for one hour. The sensitivity testing began with the left hind paw, employing a 0.6g von Frey filament. The filament was applied to the plantar surface, exerting enough force to bend the filament, and was held in contact for 1-2 seconds. The choice of the subsequent filament size was contingent on the animal’s response to the preceding one. In cases where no withdrawal response was observed, a filament with the next higher weight was used. Conversely, if a withdrawal response occurred, a filament with a lower weight was selected. This process was repeated until the paw had undergone five tests, ensuring a minimum interval of 2 minutes between each stimulus. Following this, the right hind paw was subjected to the same testing procedure (Deuis et al., 2017).

### 2.9 Statistical analyses

All statistical analyses were conducted using SPSS 22.5 (SPSS, Chicago, IL, USA), except where noted otherwise. Descriptive statistics for continuous variables are reported as means ± standard deviation (SD). The Kolmogorov-Smirnov test was used to assess the normality of data distribution. For normally distributed data from multiple groups, one-way ANOVA was employed, while the Kruskal-Wallis test was utilized for other data types. Bonferroni correction was used to adjust p-values in instances of fewer than 10 multiple comparisons, and for more extensive comparisons, p-values were adjusted using the FDR correction based on the Benjamini-Hochberg procedure. The Shannon index at the genera level was computed using QIIME (Version 1.7.0). Principle Component Analysis (PCA) was performed using FactoMineR and clusterSim packages in R software (Versuib 2.15.3). PLS-DA was performed with SIMCA-P software to cluster sample plots across different groups. A significance level α of 0.05 was set for hypothesis testing, with a p-value < α being considered statistically significant.

## 3 Results

### 3.1 Dietary fiber supplementation increases the abundance of SCFAs-producing bacterial genera

**Figure 1:**
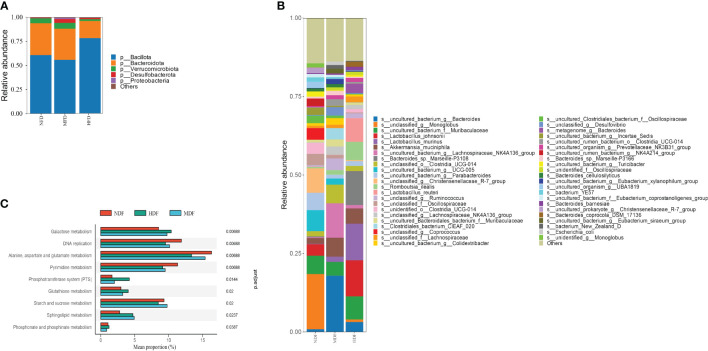
Study of microbiota by analysis of 16S rRNA sequencing. **(A, B)** A&B represent the Relative abundance at different taxa levels. The HDF group was dominated by the Bacillota phylum, whereas the NDF group was dominated by the Bacteriodota phylum analyzed through Lefse. **(C)** KEGG pathway analysis showed that the HDF group was upregulated in the galactose metabolism pathway.

In our analysis of fecal samples using 16S rRNA gene sequencing, it was observed that the MDF and HDF did not exhibit significant differences in α-diversity compared to NDF, though they showed a slight increase in relative abundance. Notably, at the phylum level, the HDF group exhibited a significantly higher abundance of Bacillota, a prominent phylum recognized for its production of butyrate, a type of SCFAs(**Figure 1A**). Additionally, employing linear discriminant analysis effect size (LEfSe) analysis, at the genus level, the prevalent genera in the HDF group were *Monoglobus*, *Muribaculaceae*, and *Clostridia*. In contrast, *Bacteroide*, *Muribaculaceae*, and *Lachnospiraceae* were the dominant genera observed in the other experimental groups(**Figure 1B**). Using KEGG annotation and functional enrichment, we identified nine distinct functional categories that exhibited varying levels of enrichment across the experimental groups. Specifically in the HDF group, functions related to galactose metabolism were notably increased (**Figure 1C**).

### 3.2 Dominant bacterial genera producing SCFAs significantly improve gut permeability

**Figure 2:**
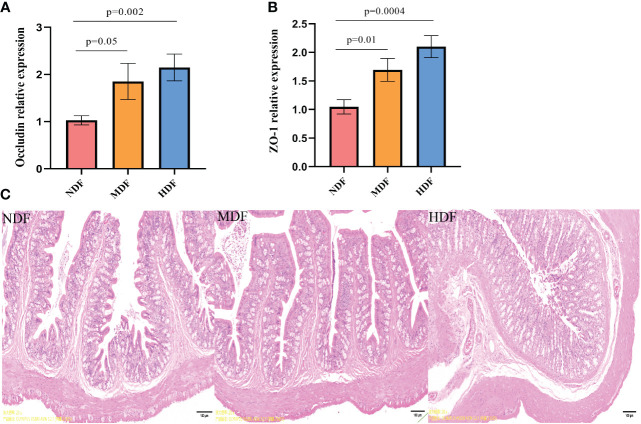
Assessment of gut permeability. Analysis of mRNA expression of tight junction protein molecules from different groups of colons by quantitative PCR methods. **(A)** Significantly increased mRNA level of tight junction protein Occludin was preserved in mice from the HDF. **(B)** Significantly increased mRNA level of tight junction protein ZO-1 was observed in mice from the HDF. **(C)** The HDF group had a thicker mucosal layer and more cup cells compared to the NDF (original magnification:4x,scale bar:100μm).

In our investigation of the changes associated with dietary fiber intake, we evaluated gut permeability. The HDF showed a significant enhancement in the expression of tight junction proteins mRNA compared to NDF (**Figures 2A, B**). Based on the findings from H&E staining, the MDF and HDF groups displayed less pronounced inflammatory cell infiltration when contrasted with the NDF (**Figure 2C**). Combining the outcomes obtained through these two approaches, we determined that the HDF group exhibited comparatively attenuated inflammation.

### 3.3 Changes in gut microbiota may affect systemic inflammation

**Figure 3:**
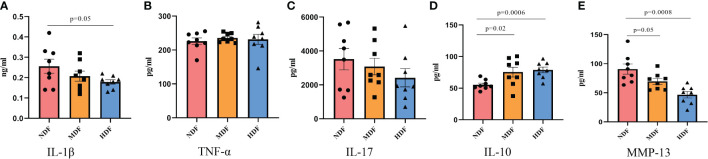
Inflammatory biomarkers analysis. The expression of systemic inflammatory factors was examined by ELISA kits. **(A)** IL-1β **(B)** TNF-α **(C)** IL-17 **(D)** IL-10 **(E)** MMP-13. (IL-1β=Interleukin 1 beta; IL-10=Interleukin 10; IL-17=Interleukin 17;TNF-α=Tumor necrosis factor α; MMP-13=Matrix Metalloproteinase 13).

The development of OA is usually accompanied by an increased inflammatory response *in vivo*, so we examined serum levels of inflammatory factors by ELISA technique. Interestingly, the findings deviated from previous observations in gut-bone axis research. Following the 8-week intervention, the levels of pro-inflammatory factors like IL-1β, TNF-α, and IL-17 in the MDF and HDF groups exhibited a downward trend, though not significantly lower than NDF (**Figures 3A-C**). However, the anti-inflammatory factor IL-10 showed a significant increase in the HDF group (**Figure 3D**). Additionally, we measured serum levels of MMP-13, a key enzyme in OA pathology known for its role in degrading collagen and other matrix molecules in joint cartilage, which contributes to cartilage damage and joint space widening. MMP-13 is thus regarded as a vital biomarker in the pathogenesis of OA. Our results indicated a significant decrease in MMP-13 expression in the HDF group compared to the NDF group, indicating a reduction in arthritis-related inflammation (**Figure 3E**).

### 3.4 Gut microbiota composition influences OA severity and SESN2 gene expression in the joint

**Figure 4:**
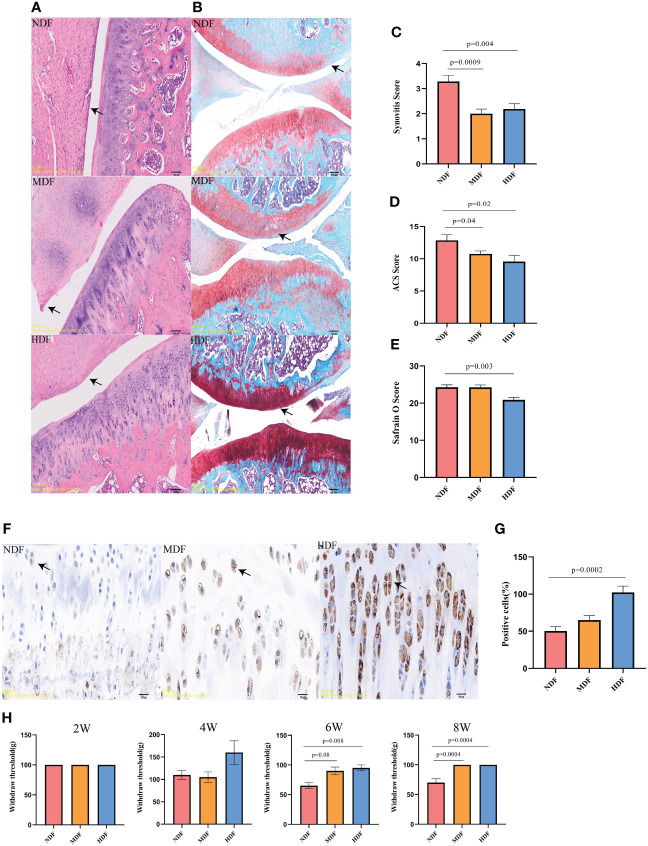
Histologic analysis of knee joints. **(A)** Representative images of H&E staining of rat knee joints between groups. (original magnification:5x). **(B)** Representative images of panchromatic solid green-stained sections of rat knee joints between groups(original magnification:5x). **(C)** Synovitis scores between groups. **(D)** Safranin O scores between groups. **(E)** ACS scores between groups. **(F)** Representative images of SESN2 immunohistochemistry at the knee joints of different groups of rats, (original magnification: 20x). **(G)** Number of positive cells between groups. Note: brown particles in the Figure are SESN2 positive chondrocytes. (Scale bar in the above image, 100μm) **(H)** Von Frey test results in rats at different time points. Each bar represents a group (x-axis) with mean and 95% confidence interval (y-axis). The black solid line is a reference to the mean histologic score of the rats.

To comprehensively evaluate knee joint lesions, our evaluation encompassed three distinct methods: H&E staining, Safranin-O staining, and immunohistochemical.We found that the HDF group demonstrated significantly lower synovitis scores compared to the NDF group, aligning closely with the MDF group’s results (**Figures 4A, C**). In evaluating cartilage damage, the NDF group showed higher scores in both Safranin-O and ACS scoring, indicative of more extensive damage compared to the groups receiving fiber supplementation (**Figures 4B, D, E**). The HDF group was distinguished by significantly higher expression of SESN2 protein compared to the other two groups, showing a significant difference from the NDF (**Figures 4F, G**). In terms of pain assessment, following the six-week intervention period, the withdrawal thresholds of the MDF and HDF groups were significantly higher than those of the control group and the two groups became similar at 8 weeks (**Figure 4H**). These observations suggest that the HDF group’s dietary regimen played a substantial role in slowing the progression of OA, potentially linked to the upregulated expression of SESN2.

## 4 Discussion

OA, one of the culprits affecting the quality of life of most people, is increasing in prevalence every year. In the process of exploring the mechanisms of OA, we have found that aging and weight gain are very important influencing factors. And existing studies on the gut-bone axis show that aging and obesity of the organism have a significant impact on the composition of the gut microbiota. Dietary fiber, an easily overlooked nutrient, has been shown in most studies to have a positive effect on the health of the organism. Studies have confirmed that a certain amount of dietary fiber intake can help slow down aging or reduce the risk of obesity (Matt, 2018; Delzenne, 2020). According to epidemiological surveys, the daily intake of dietary fiber should be 25-28g, but the irregular lifestyle has resulted in the inadequate intake of dietary fiber (Gill et al., 2020). Our study delves into the health benefits of dietary fiber and helps to raise awareness of the need for dietary fiber intake.

We observed that a diet rich in dietary fiber induced significant changes in the gut microbiota. The diversity of the gut microbiota exhibited no statistically significant variance between the two groups. Instead, the primary differences emerged in the abundance of dominant genera within the respective groups. Specifically, in the HDF, the gut microbiota predominantly comprised Bacillota, whereas the gut microbiota of the NDF was primarily characterized by an abundance of Bacteriodota. Notably, as early as 1986, research had unveiled distinctions in the types of SCFAs produced by these bacterial groups (Macfarla, 1986). Bacillota were found to predominantly generate butyric acid, while Bacteriodota were observed to predominantly produce acetic and propionic acid (Macfarlane and Macfarlane, 2003; Feng et al., 2018). The composition of the dominant genera in the body varies, as does the permeability of the constructed intestinal barrier as well as its immunological capacity. In recent studies in Inflammatory bowel diseases (IBD), there are results suggesting that an increase in butyrate production may improve the integrity of the colonic epithelium and the immune function of the mucosa to a certain extent, resulting in, among other things, anti-inflammatory effects (Wu, 2021). This result aids in confirming that the addition of SCFAs is relevant for maintaining intestinal integrity.

Furthermore, the high fiber intake positively impacted the initial stages of synovial inflammation, mitigated cartilage damage, boosted chondrocyte activity, and alleviated the perception of knee joint pain. At the conclusion of the 8-week trial, a notable increase in SESN2 gene expression was observed in the knee joints of the HDF group, primarily due to differences in the dominant genera of gut microbiota. This finding aligns with a prior study that compared SESN2 expression in normal, elderly, and osteoarthritic joints, revealing significantly lower SESN2 expression in aging osteoarthritic joints compared to normal conditions. The SESN2 protein plays a pivotal role in cellular stress responses and the maintenance of redox homeostasis. Its influence extends to crucial aspects of cell biology, encompassing cell survival, proliferation, and function. Notably, alterations in the gut microbiota composition have the potential to modulate SESN2 gene expression. A study from 2018 conducted at the Scripps Research Institute demonstrated that SESN2 can activate the mTOR pathway through autophagy, thereby contributing to the preservation of chondrocyte activity (Shen et al., 2017). Thus, it becomes evident that the composition of gut microbiota exerts a significant impact on the development and severity of OA. Our findings not only furnish a theoretical basis for further investigations but also kindle substantial interest in exploring the functions of SESN2 and its interplay with the gut microbiota in the context of arthritis. Our joint analysis revealed notable findings: the HDF group, characterized by an abundance of the Bacillota phylum, significantly upregulated the expression of SESN2, diminished inflammation in the OA joint, and exhibited less severe cartilage damage. This contrasted with the NDF group, which displayed the opposite trend. While previous research has shown that butyrate, a fermentation product of gut microbiota, enhances SESN2 expression in the liver, its influence on arthritis progression has not been explored. Our study fills this gap by demonstrating that a Bacillota-dominant microbiota profile is associated with increased SESN2 expression in the knee joint. Crucially, this elevation in SESN2 expression correlates with reduced synovial inflammation and chondrocyte damage, suggesting a potentially protective and therapeutic role in OA progression and management.

The early fiber intervention studies found that it could increase the level of SCFAs in the body and also down-regulate the expression of pro-inflammatory chemokines MCP-1, IL-18 and IL-33, to the direct supplementation of SCFAs, which was shown to regulate inflammation and metabolism through two signaling pathways, namely, GPCRs and HDACs (Durholz et al., 2020; He et al., 2020). In addition, in rheumatoid arthritis, SCFAs have been shown to regulate B-cell differentiation through FFA2 receptors to alleviate rheumatoid arthritis (Yao et al., 2022). Numerous studies have demonstrated that the addition of dietary fiber can improve inflammation in the body to a certain extent, but there is a gap in the direction of how different levels of dietary fiber can improve inflammation. Unlike previous studies, this study compared the level of beneficial microorganisms produced by different levels of dietary fiber and the difference in the degree of improvement of OA, which provides data support for further research on the composition of the gut microbiota and the changes in OA after dietary fiber supplementation. Cohort studies on dietary fiber have also demonstrated the importance of dietary fiber for OA improvement from different perspectives. Dr. Dai et al. demonstrated a strong correlation between high fiber intake and varying degrees of OA pain in an 8-year clinical investigation (Dai et al., 2017). A Canadian longitudinal study has also shown that a certain level of high fiber intake is necessary to build a better body composition, which is beneficial in combating the development of OA (Chopp-Hurley et al., 2022). The analysis of OA changes in different dietary patterns further demonstrates the importance of the body’s inflammatory cytokines as targets (Zeng et al., 2023). While it is important to understand the benefits of dietary fiber intake, the health effects of dietary fiber deficiency cannot be ignored. For example, in 2016, microbial communities were artificially created in germ-free mice, and it was found that in the absence of dietary fiber, the colonic mucus barrier in the mice was severely disrupted, favoring the invasion of foreign pathogens (Desai et al., 2016). On this basis, we found that prebiotic dietary fiber deficiency predisposes to a high tensinogenic gut microbiota, the composition of which reduces the production of SCFAs and inhibits the GPR43/109A signaling pathway, thereby contributing to the progression of cardiovascular disease (Kay, 2020). Similarly, in neurodegenerative diseases, fiber deficiency has been shown to accelerate disease progression by altering the composition of the gut microbiota (Shi et al., 2020).

The link between dietary fiber and OA is strong, and the investigation of the molecular mechanisms between the two is particularly urgent. A notable strength of this study lies in its pioneering approach of comparing changes in dominant bacterial taxa within the gut microbiota in response to various levels of dietary fiber. This approach stands out for its health-centric and practical nature, especially when contrasted with approaches that involve artificial supplementation with other health products. Our study, while offering significant insights, still has certain limitations. Although rats and other animals are often used as model animals for research purposes, the applicability of these animal data to humans remains an open question. We will build on the existing studies to further identify the specific types of SCFAs that promote the upregulation of SESN2. Future research should aim to explore cellular mechanisms to clarify how different SCFAs affect chondrocyte activity. This will provide more precise and actionable information for clinical practitioners in treating patients.

## 5 Conclusions

Our study demonstrated that dietary fiber supplementation can change gut microbiota composition and slow the progression of OA. We observed a strong correlation between the abundance of the Bacillota phylum in the gut microbiota and OA severity, which was also associated with changes in SESN2 expression. These observations indicate a potential role for Bacillota in ameliorating OA, possibly mediated through SESN2. Our results support the concept of modulating gut microbiota through dietary interventions as a promising therapeutic approach for OA. Future research endeavors should aim to elucidate the underlying mechanisms of SESN2 and explore how these findings can be effectively translated into clinical practice.

## Data availability statement

The datasets presented in this study can be found in online repositories. The names of the repository/repositories and accession number(s) can be found below: NCBI, PRJNA1097012.

## Ethics statement

The animal study was approved by Animal Care and Use Committee of Jiangnan University. The study was conducted in accordance with the local legislation and institutional requirements.

## Author contributions

YWu: Conceptualization, Data curation, Investigation, Methodology, Project administration, Resources, Software, Supervision, Validation, Writing – original draft, Writing – review & editing. XL: Conceptualization, Data curation, Investigation, Methodology, Resources, Validation, Writing – original draft, Project administration. HM: Conceptualization, Investigation, Methodology, Supervision, Validation, Writing – original draft, Data curation. YWa: Conceptualization, Investigation, Methodology, Project administration, Supervision, Writing – original draft. PS: Conceptualization, Data curation, Investigation, Supervision, Validation, Writing – original draft. YD: Conceptualization, Investigation, Supervision, Validation, Writing – original draft. JY: Conceptualization, Data curation, Investigation, Supervision, Validation, Writing – original draft. BC: Conceptualization, Data curation, Investigation, Methodology, Project administration, Resources, Supervision, Validation, Writing – review & editing. XW: Conceptualization, Data curation, Investigation, Validation, Writing – original draft, Writing – review & editing, Funding acquisition, Methodology, Project administration.
