# Supplementary material for: Dietary fiber may benefit chondrocyte activity maintenance
Source: Front Cell Infect Microbiol. 2024 May 13;14:1401963. doi: 10.3389/fcimb.2024.1401963 (PMC11129558; doi:10.3389/fcimb.2024.1401963)
Supplement: Supplementary file 1 [file DataSheet_1.docx]

Supplementary Material

# Supplementary Data

16Sr RNA gene sequencing and statistical analysis

Microbial DNA was extracted from fecal samples using an E.Z.N.A.® soil DNA Kit (Omega Bio-tek, Norcross, GA), according to the manufacturer ’s protocols. In Brief, The NanoDrop 2000 UV-vis spectrophotometer (Thermo Scientific,Wilmington, Delaware, United States) was used to determine the final DNA concentration and purity, and DNA quality was checked using 1% agarose gel electrophoresis.Variable regions 3 and 4 (V3-V4) of bacterial 16S-rRNA were amplified using 338F_806R barcode primers. The PCR product was extracted from 2% agarose gel and purified using an AxyPrep DNA Gel Extraction Kit (Axygen Biosciences, Union City, CA), according to the manufacturer’s instructions, and quantified using Qubit 4 (Thermo Fisher, United States). Purified amplicons were pooled in equimolar and paired-end sequenced on the Illumina MiSeq PE300 platform (Illumina, SanDiego, United States ) according to the standard protocols by Honsunbio Technology Co. Ltd (Shanghai, China). Bioinformatics analysis Sequencing reads were demultiplexed, quality controlled by fastp (version 0.21.0), and merged by FLASH (version 1.2.7).

Shortly, reads with adaptor sequences and low quality bases (quality score <Q20) were trimmed. Truncated reads shorter than 50 bp and reads containing ambiguous nucleotides were discarded. Subsequently, the paired-end reads were merged according to the minimum overlap of 10 bp with maximum mismatch ratio of 0.2 in the overlapping region. Only merged sequences were retained for downstream analyses. The UPARSE algorithm was used to cluster sequences with a 97% similarity cutoff, while chimeric sequences were identified and removed. Next, the taxonomy of each OTU representative sequence was assigned by using RDP Classifier against the reference database SILVA138 with a minimum confidence score of 0.7. Rarefaction was performed in order to compare the abundance of OTUs across samples. Sequences demultiplexed were imported to QIIME2 (version 2022.8). The DADA2 algorithm was used to quality filter and denoise sequences, while chimeric sequences were removed. Next, the taxonomy of each ASVrepresentative sequence was assigned by using RDP Classifier against the reference database SILVA138 with a minimum confidence score of 0.7. The numberof sequences from each sample was normalized to the lowest number of read counts by randomly selecting subsets of sequences. Statistical analysis The R （version 4.1.3） was used to perform general statistical analysis and visualize results via packages vegan (v2.6-4), phyloseq (v1.38.0), tidyverse (v1.3.2), ggpubr (v0.5.0), ComplexHeatmap (v2.10.0) and corrplot (v0.92). Alpha diversity was estimated using the Sobs, ACE, Chao1, Shannon and Simpson indices. Principal coordinates analysis (PCoA) based on bray-curtis matriceswith statistical significance determined by permutational multivariate analysis ofvariance (PERMANOVA) was conducted to assess the differences in beta diversity between groups. For comparing the relative abundance of different taxa between groups,linear discriminant analysis (LDA) effect size (LEfSe) method wasperformed with a p-value < 0.05 for the Kruskal–Wallis test and a size-effect threshold of 2.0 on the logarithmic LDA score. Spearman ’ s rank correlation analysis was used for correlation analysis.
